# Monitoring the outcomes of interventions against *Taenia solium*: options and suggestions

**DOI:** 10.1111/pim.12291

**Published:** 2016-03-08

**Authors:** M. W. Lightowlers, H. H. Garcia, C. G. Gauci, M. Donadeu, B. Abela‐Ridder

**Affiliations:** ^1^ Veterinary Clinical CentreFaculty of Veterinary and Agricultural SciencesThe University of MelbourneWerribeeVic.Australia; ^2^ Cysticercosis UnitInstituto Nacional de Ciencias NeurológicasLimaPeru; ^3^ Department of MicrobiologySchool of SciencesCentre for Global Health–TumbesUniversidad Peruana Cayetano HerediaLimaPeru; ^4^ Department of Control of Neglected Tropical DiseasesWorld Health OrganizationGenève 27Switzerland

**Keywords:** control, cysticercosis, diagnosis, neurocysticercosis, *Taenia solium*, taeniasis

## Abstract

There is an increasing interest in reducing the incidence of human neurocysticercosis, caused by infection with the larval stage of *Taenia solium*. Several intervention trials are currently assessing various options for control of *T. solium* transmission. A critical aspect of these trials will be the evaluation of whether the interventions have been successful. However, there is no consensus about the most appropriate or valuable methods that should be used. Here, we undertake a critical assessment of the diagnostic tests which are currently available for human *T. solium* taeniasis and human and porcine cysticercosis, as well as their suitability for evaluation of intervention trial outcomes. Suggestions are made about which of the measures that are available for evaluation of *T. solium* interventions would be most suitable, and which methodologies are the most appropriate given currently available technologies. Suggestions are also made in relation to the most urgent research needs in order to address deficiencies in current diagnostic methods.

## Introduction

*Taenia solium* is a cestode parasite transmitted between humans acting as definitive hosts and pigs as intermediate hosts. The medical significance of the parasite arises not from it causing taeniasis but because humans may become infected with the larval or metacestode life cycle stage, in which case it commonly encysts in the brain and spinal cord, causing neurocysticercosis.

Neurocysticercosis is a major cause of neurological disease in many developing countries 1, 2 and an increasing cause of disease in the developed world due to migration of presymptomatic neurocysticercosis patients 3, 4. In endemic areas, neurocysticercosis is considered to be the most frequent preventable cause of seizure disorders, being associated with 29% of people with epilepsy 5. *T. solium* is ranked as the most important foodborne parasitic infection from a global perspective 6. The World Health Organization added infections with *T. solium* to the list of Neglected Tropical Diseases in 2010 and continues to actively promote the development and implementation of strategies to decrease the incidence of neurocysticercosis 7.

The full lifecycle of *T. solium* is only perpetuated where pigs eat human faeces, or eat items contaminated by human faeces. For this reason, full transmission of the parasite from humans to pigs and back to humans occurs where sanitation is poor, meat inspection is limited or missing, awareness is low and facilities for safe preparation of food are inadequate. These conditions are reflected in poor, neglected populations; there is no demand for control of the parasite's full transmission cycle in developed countries. This places a considerable constraint on the methods that would be feasible or sustainable to achieve control of the parasite's transmission.

Consideration of the life cycle of *T. solium* immediately identifies several potential opportunities where parasite transmission could be interrupted: treatment of human taeniasis carriers to remove adult tapeworms, education of the population about personal hygiene to prevent human‐to‐human transmission and safe disposal of human faeces, preventing pigs gaining access to human faeces or items contaminated with faeces, meat inspection and removal of contaminated meat from the food chain, treatment of pigs to kill muscle cysticerci or vaccination of pigs to prevent the establishment of cysticerci. Several experimental trials have been undertaken to control *T. solium* transmission by implementing one or a combination of these strategies 8, 9. Results of these trials have been variable; however, to this time, there are few examples where a substantial level of control has been achieved and has endured.

Three relatively new tools are available to assist in the control of *T. solium* – coproantigen testing for taeniasis 10, treatment of pigs with oxfendazole to kill cysticerci 11 and the TSOL18 vaccine for pigs to prevent *T. solium* infection 12, 13, 14. There is an increasing interest in implementing a One Health approach to human and animal diseases, and this applies most obviously to zoonotic infections such as *T. solium*. A growing awareness of the need to evaluate existing strategies to control *T. solium* is stimulating new initiatives to evaluate intervention options that span different sectors.

An obvious and important aspect of any intervention against *T. solium* is an evaluation of the outcomes of control initiatives. Not so obvious is which outcomes can or should be measured. The overall objective of *T. solium* control is to reduce the incidence of human cysticercosis. Other, indirect measures that would reflect changes in *T. solium* transmission are the incidence of human taeniasis due to *T. solium* or the incidence of porcine cysticercosis, changes in which could be expected to affect, directly or indirectly, the risk for new cases of cysticercosis in humans. Each of these different aspects of the *T. solium* life cycle can be assessed by a variety of techniques. Currently, there is no consensus about which *T. solium* assessment measures would be most suitable or effective for evaluating cysticercosis control initiatives. One of the most important attributes of any measure that is used to assess *T. solium* control efforts is specificity. Evaluation methods that have poor specificity for *T. solium* risk a misinterpretation of the outcomes of control activities because each false positive in a test would imply a control failure. Some tests for *T. solium* infection are either known to have poor specificity or they have been inadequately assessed in this respect. Another vital factor that affects the choice of assessment measures is their availability, particularly in endemic countries, and the feasibility of tests due to cost or technical complexity.

In the following review, we undertake a critical assessment of the tests that are potentially available to monitor and evaluate the outcomes of *T. solium* control. The review is not intended to be comprehensive, rather it focuses on assessment methods that have been, or are being used to monitor *T. solium* interventions, and those supported by verified data. Nor is it intended to evaluate the diagnostic tests used for the individual human patient. Comments are made about what are considered to be the most urgent research or development requirements needed to improve the effectiveness or availability of *T. solium* assessment tools and evidence‐based suggestions are made about which tests should be used to monitor *T. solium* interventions, based on current knowledge.

## Human Cysticercosis/Neurocysticercosis

Untreated cases of *T. solium* cysticercosis in humans may endure for many years, and for this reason, the prevalence of human cysticercosis would not be an appropriate measure to evaluate the outcomes of anything other than long‐term interventions. Assessment of changes in the incidence of cysticercosis in the human population would be meaningful, but only if control measures were being implemented so as to affect a sufficiently large population to allow significant changes to be detected, and over a sufficient interval of time. Until now, those interventions for *T. solium* that have attempted to assess changes in the level of human infections have used serological methods or questionnaires that were not necessarily able to confirm infection *per se*. In a clinical setting, neurocysticercosis is diagnosed using a combination of imaging techniques together with serology for confirmation 15. Sufficient resources are unlikely to be available to use imaging for the detection of neurocysticercosis on a population basis, other than possibly in exceptional circumstances.

### Antibody detection tests

Several serological tests have been developed for the detection of the presence of anti‐*T. solium* antibodies induced by cysticercosis in humans and are considered to have a high level of specificity 16. Parkhouse and Harrison 17 applied lentil lectin affinity chromatography for the identification of *T. solium* cysticercus glycoprotein antigens recognized by patients with cysticercosis. Tsang *et al*. 18 utilized similar methods in a Western immunoblot assay (designated enzyme‐linked immunoelectrotransfer blot or EITB) and developed a method considered by the authors to have absolute specificity for *T. solium* 16, 18. The antigens recognized in the test have been characterized and produced as synthetic or recombinant proteins 19. Attempts to date to convert the test from a Western blot assay to an ELISA format have not resulted in sensitivity/specificity outcomes comparable with that achieved using native antigens 20, 21. Ito *et al*. 22 developed an ELISA‐based assay using cysticercus antigens purified by preparative isoelectric‐focusing. The test shows a high sensitivity and specificity for diagnosis of human cysticercosis but has not been widely adopted, possibly because of the resources required to prepare the antigen.

Several serological tests for human cysticercosis are widely available as commercial products. Some manufacturers of ELISA kits mention limitations due to cross‐reactions with *Echinococcus*. Prices vary widely, from US$ 5 to 30 per test depending on the manufacturer and the supplier. Tests based on Western blots (EITB) are available commercially but the price is even higher (up to US$ 347/sample for the test provided by one company). Tests for *T. solium*‐specific antibodies do not differentiate viable from nonviable infections.

Garcia *et al*. 23 undertook a longitudinal study of the serological reactivity to *T. solium* in people living in three different disease‐endemic areas of Peru and Colombia. The study revealed that many people display transient but unequivocally positive reactions in anti‐*T. solium* antibody detection tests, in the absence of any other evidence to suggest that the people harboured detectable cysticerci. These reactions may have been due to exposure to *T. solium* (eggs) that did not lead to the establishment of a continuing, detectable infection with cysticerci. This interpretation is supported by substantial evidence indicating that the EITB shows very high specificity for *T. solium* 16, 18. The transient nature of some serological responses to cysticercosis antigens was confirmed by Meza‐Lucas *et al*. 24 in Mexico and more recently using EITB with sera from residents in an endemic region of Zambia 25. However, there is no direct evidence to support an association between *T. solium* exposure in humans and transient positive reactions in EITB. In pigs, cross‐reactions were found in *T. solium* EITB when sera were tested from specific pathogen free animals that had been exposed to *Taenia asiatica* 26, highlighting the potential for false‐positive reactions possibly occurring in humans exposed to *Taenia* sp. other than *T. solium*. Further evidence is needed to confirm that transient positive reactions in anti‐*T. solium* antibody detection tests are uniquely associated with exposure to *T. solium*.

### Antigen detection tests

Several serological tests have been developed which detect circulating *T. solium* cyst antigens in humans 16. The two most commonly evaluated tests were both originally developed to detect infection with *Taenia saginata* in cattle 27, 28, 29, and both were subsequently found to detect *T. solium* cysticercosis in pigs 30, 31 and humans 32, 33. One antigen ELISA for *T. solium* is available commercially (apDia, Belgium); the current cost of the test (€145 per kit, duplicate testing, cost €3·15/sample) would be a significant disincentive to its use on a population‐wide basis. Patient's sera are only positive in *T. solium* antigen ELISA in cases where there is infection with viable *T. solium* cysticerci 32, 33. Antigen ELISA has been described as being sensitive for *T. solium* infections in humans where there are ≥2 viable cysts 34; little information is available about the performance of the commercially available test. Analysis of sera from humans with parasitic infections, or conditions other than *T. solium* infection, indicates the test is highly specific for *T. solium* 16, 33.

Two recent studies undertaken in Zambia 25 and Ecuador 35 have revealed that many antigen ELISA‐positive patients among people living in areas that are endemic for transmission of *T. solium* have only transient positive reactions and are likely to revert to being seronegative. These responses may have been due to infections that never fully establish but led to transient antibody responses and the short‐term presence of circulating antigen. However, as discussed above in relation to antibody detection tests, there is no direct evidence to support this view. The communities in Zambia 25 and Ecuador 35 where longitudinal studies were undertaken using antigen ELISA would be likely to have been endemic for transmission of several taeniid cestode parasites involving humans, dogs or other carnivores as definitive hosts, not just *T. solium*. There is potential for exposure of humans to the eggs of a number of these species, any of which could possibly cause a transient immunological response detected in the *T. solium* antigen ELISA. It is known that infection with either *Taenia hydatigena* 36 or *T. asiatica* (a parasite most closely related to *T. saginata*) 37 in pigs leads to persistent strong positive reactions in the same *T. solium* antigen ELISA. Transient positive responses in the *T. solium* antigen ELISA in humans could possibly be due to exposure to eggs of *T. hydatigena* or *T. saginata,* or of other taeniid cestode species, that did not lead to actual infections.

### Research/development needs

- Additional evidence is needed to determine whether transient positive responses seen in anti‐*T. solium* antibody or serum antigen tests in people living in endemic areas are due to exposure to *T. solium* eggs.
- Refinement of both the EITB and antigen ELISA methodology to provide simpler and less expensive procedures.

## *Taenia Solium* Taeniasis

It seems intuitively obvious that monitoring of *T. solium* taeniasis would be of value for assessing the outcomes of *T. solium* control activities; human taeniasis is the sole source of human cysticercosis. A decline in, or absence of, *T. solium* taeniasis would be reflected in a reduced risk of new cases of cysticercosis. However, whether it would be meaningful and valuable to invest resources in diagnosis of taeniasis would depend on the prevalence of *T. solium* taeniasis at the outset of an intervention, the size of the population that would be affected by the intervention and the sensitivity and specificity of the tests available for the detection of *T. solium* taeniasis.

Little accurate information is available about the prevalence of *T. solium* taeniasis in *T. solium* endemic areas of the world because most of the available methods to diagnose *T. solium* taeniasis are lacking in either sensitivity or both sensitivity and specificity. It is considered that the prevalence of *T. solium* taeniasis is commonly 1–2% of the population in many endemic regions 2 although substantially higher rates have been reported for some areas 38, 39.

### Direct assessment of taeniasis cases

One option for monitoring *T. solium* interventions is to determine the number of patients with *T. solium* taeniasis in the community following mass treatment with taeniacide. This could be undertaken even if treatment of taeniasis were not a formal component of an intervention, for example in the case where intervention was undertaken by public education alone or by interventions in pigs alone. Species‐specific identification of *T. solium* tapeworms would need to be carried out on worms voided by taeniasis carriers after treatment. A systematic collection scheme would be required to collect the worms, and a post‐treatment screen of stools, after mass treatment; otherwise, only a very small proportion of worms would be likely to be recovered.

### Microscopic examination of faeces for *Taenia* eggs

Microscopic examination of faecal samples for the presence of *Taenia* eggs has been used frequently in epidemiological studies of taeniasis 40, 41, 42. Irrespective of the particular technique used for the examination, it is known to be relatively insensitive 43, 44. Hall *et al*. 44 were successful in diagnosing taeniasis due to *T. saginata* in 68% of confirmed cases, and Li *et al*. 40 detected as few as 51·5% of cases of *T. solium* or *T. saginata* taeniasis by faecal microscopy. In settings of a low prevalence of infection, the cost/benefit ratio of this method of assessment would be low.

Coprology may identify the presence of a *Taenia* tapeworm; however, detection of eggs by microscopy will not itself determine which species is present because the eggs of different *Taenia* species are indistinguishable. Where mature proglottids are identified, they can be differentiated between *T. solium* and *T. saginata* on morphological criteria. Species‐specific diagnosis would be irrelevant if the purpose of the testing were to identify individuals so they could be treated because treatment would be warranted in any case. However, diagnosis to species level would certainly be necessary when data about taeniasis were to be used to monitor the outcomes of *T. solium* interventions; otherwise, infections with *T. saginata* or *T. asiatica* would confound the impact of control efforts specifically on *T. solium*.

### Coproantigen detection assays

Parasite products other than eggs or proglottids are released with the faeces of *Taenia* carriers, and these can be detected in faecal samples using antibodies raised against excreted/secreted and/or adult somatic antigens. The tests are commonly known as coproantigen detection or CoproELISA. The first coproantigen test for taeniasis was devised by Allan *et al*. 10. It detected the presence of a *Taenia* sp. tapeworm and, although it was not species specific for *T. solium*, it had a higher sensitivity than faecal microscopy for diagnosis of taeniasis 45. Guezala *et al*. 46 used antisera raised against *T. solium* adult excretory–secretory (ES) antigens together with a capture antibody raised against adult somatic antigens in a coproantigen ELISA and were able to differentiate *T. solium* taeniasis from *T. saginata*. Although reproduced by the authors, no subsequent publications refer to use of this species‐specific test and the reagents are no longer available (PS Craig, personal communication).

One area for concern regarding coproantigen tests is their reliance on polyclonal antisera. Different batches of polyclonal antisera are likely to have different titres and a different spectrum of antigen specificities. In replicating a published technique that relies on polyclonal antisera, it cannot be assumed that the replicated test would have an equivalent performance in regard to sensitivity and specificity for diagnosis of taeniasis to that described by other groups. Every new batch of reagent, and every new group that wished to adopt the method, would need to validate their reagents for sensitivity and specificity. Unfortunately, this has not occurred and coproantigen testing has been used by a number of researchers without providing adequate data to validate the performance of their particular variation of the test. All coproantigen tests rely on the use of specialized reagents and are currently not commercially available, limiting the use of coproantigen testing to research studies. Nevertheless, coproantigen testing has proved valuable in some studies. Bustos *et al*. 47 identified patients with taeniasis in which treatment with niclosamide had failed, showing that 89% of successfully treated patients were coproantigen ELISA negative after 7 days, while 94·1% of treatment failures were coproantigen ELISA positive after 7 days. An interesting recent discovery has been the finding that the coproantigen ELISA test is not reliably positive until the tapeworm is, or nears being, gravid 48. The observation was made by Tembo and Craig 48 while assessing the faeces of an individual after self‐infection with *T. saginata*.

### Detection of specific antibody in serum

Following the discovery in 1985 that animal definitive hosts of *Taenia* sp tapeworms induce specific antibodies that can be detected in serum 49, 50, it was found that the same was also the case for human taeniasis 51. The antigens that were found to be useful for serological diagnosis of *T. solium* taeniasis were ES products collected from adult tapeworms 51. Levine *et al*. 52, 53 cloned and expressed two of the antigens, designated ES33 and ES38, and evaluated them for diagnosis of *T. solium* taeniasis. Both were very effective for diagnosis of *T. solium* taeniasis in an EITB format. The results were promising, with rES33 and rES38 having sensitivity/specificities of 98%/99% and 99%/97%, respectively. rES33 was better able to discriminate *T. solium* from *T. saginata* taeniasis 52. The antigens have subsequently been evaluated in studies investigating two user‐friendly test formats: multi‐antigen print immunoassay and a magnetic immunochromatographic test 54, 55. In these formats, some degree of cross‐reactivity was evident among sera from patients with cystic echinococcosis (5% cross‐reacting) and schistosomiasis (14–17%).

In circumstances where taeniasis was to be monitored as part of a *T. solium* control effort, and where the starting prevalence of taeniasis was not more than 2%, serological tests for taeniasis would necessarily be required to have a level of false‐positive reactivity in the target population of less than 2%; otherwise, the majority of the cases detected would be false positives. The serological test for taeniasis developed by Levine *et al*. 52, 53 may be valuable as a screening assay in *T. solium* control programs, but positive cases would need to be verified by other means.

One area of concern in relation to using tests for the detection of specific circulating antibodies in patients with taeniasis is that the antibodies could possibly remain detectable for some time after individuals have been treated and were no longer infected. The persistence of specific antibodies to adult tapeworms following treatment for taeniasis has not been evaluated.

### DNA‐based methods

Numerous methods have been developed which can identify, specifically, the presence of *T. solium* DNA using PCR‐based technologies. Many of these methods have been developed and applied to DNA purified directly from tapeworm or other parasite materials [reviewed in 56]. A smaller number of PCR‐based methods have been developed and validated for the detection of *T. solium* DNA with faecal material from patients with taeniasis who were subsequently proven to have been infected with *T. solium* 57, 58, 59, 60.

Where PCR‐based methods are used to differentiate *T. solium* proglottids from those of other *Taenia* species, they have distinct advantages over the use of morphological characteristics (number of uterine branches in proglottids or other characteristics) as the latter requires both specialist knowledge and skills. However, the more substantial value of PCR‐based methods is their potential to diagnose taeniasis using only a faecal sample.

PCR‐based methods are able to identify a higher proportion of taeniasis cases than are able to be diagnosed by microscopy alone, and a combination of both microscopy and coproPCR further improves diagnostic sensitivity (some proven egg‐positive cases are negative by coproPCR 59). Specificity for coproPCR is high with control faecal samples, including samples from patients with other parasitic infections, being almost always negative in coproPCR 57, 58, 59, 60, 61.

Yamasaki *et al*. 59 established a multiplex PCR for differential diagnosis of taeniasis using amplification of the gene encoding cytochrome c oxidase subunit I (cox1). This approach has particular value in areas where *T. solium*,* T. saginata* and *T. asiatica* occur, as it allows differentiation between all three species. The method was assessed in a community level study in which 92 of 132 (69·7%) tapeworm carriers, confirmed by tapeworm or proglottid expulsion, were correctly diagnosed by coproPCR 40. Combined with microscopy, the positive detection rate increased to 102 of 132 (77·3%). Ten of the 132 confirmed patients with taeniasis that were egg positive by microscopy were negative using PCR.

Praet *et al*. 60 developed a real‐time multiplex PCR for diagnosis of taeniasis which amplifies part of the internal transcribed spacer 1 of the ribosomal RNA. The protocol was applied to DNA extracts from 23 Ecuadorian stool samples which had previously been shown to be *Taenia* egg positive by microscopy. While all 23 faecal samples were PCR positive for the presence of *T. solium* and/or *T. saginata* DNA, it appears that the diagnoses were not confirmed to species level parasitologically. It is not clear what sensitivity the test would have for detecting taeniasis if it were applied to faecal samples that had not been preselected as being egg positive by microscopy.

Mayta *et al*. 57 developed a nested PCR approach utilizing two rounds of PCR amplification of the Tso31 gene. Although the nested PCR approach imposes technical disadvantages, it has the advantage of having improved sensitivity. Mayta *et al*. 57 were able to detect as few as 40 *T. solium* eggs per gram of faecal material, although it is unclear what quantum of eggs could be expected to be found per gram of a taeniasis patient's faeces. In 32 stool samples from *T. solium* carriers, 97% were found to be positive, and specificity was 100% when tested using 123 stool samples positive for other parasites including *T. saginata*,* Ascaris lumbricoides*,* Hymenolepis sp*., *Diphyllobothrium sp*. and hookworms. These are promising results, and the test warrants further validation.

Technical complexity and cost are disadvantages of the use of coproPCR as a mass screening tool for taeniasis in endemic areas. Other challenges include difficulties in the extraction of DNA from faeces and the presence of PCR enzyme inhibitors in faeces 62. Nkouawa *et al*. 58 applied a DNA‐based test known as loop‐mediated isothermal amplification (LAMP) to address some of these challenges. The method provides species differentiation and does not require sophisticated equipment 63. Using the LAMP method and amplifying the cox1 gene, Nkouawa *et al*. 58 were able to differentially identify *T. saginata*,* T. solium* and *T. asiatica* in 37 of 43 (86%) faecal samples from patients with parasitologically confirmed taeniasis. Data concerning specificity of the test are limited, although DNA prepared from parasite tissues of *A. lumbricoides*,* Enterobius vermicularis, Hymenolepis nana* and hookworms were shown to be negative. The method has been applied to a field survey, although it was only used on DNA extracted from proglottids rather than faecal samples 63. The test appeared to be effective even in very low technology conditions (no thermocycler) and warrants further investigation and validation.

In conclusion, the DNA‐based methodology developed by Mayta *et al*. 57 provides the highest sensitivity and specificity of those methods that have been developed and validated using unselected faecal samples from parasitologically proven taeniasis carriers. While the method is technically challenging and could be expected to be relatively expensive to use, the reagents required are available worldwide and laboratories capable of undertaking PCR competently are present in most countries and in many regional areas.

### Research/development needs

- Further and independent validation of the nested PCR method developed by Mayta *et al*. 57.
- Further and independent validation of the LAMP methodology described by Nkouawa *et al*. 58, especially validation of the test's potential to be effective when undertaken outside a sophisticated laboratory environment.

## Porcine Cysticercosis

While there is no doubt that eating infected pig meat is the major cause of *T. solium* taeniasis in humans, in some circumstances dogs may act as intermediate hosts for transmission of *T. solium* and may contribute to disease transmission where dog meat is consumed 64, 65. Reductions in porcine cysticercosis could be expected to be reflected in a reduced risk for human *T. solium* taeniasis and hence human cysticercosis. Monitoring porcine cysticercosis has been the most frequently used method for evaluation of *T. solium* control activities. It is easier and cheaper than testing humans as it does not involve the same level of expertise and/or ethical considerations. A major advantage of monitoring porcine cysticercosis over human infections is the much shorter life span of pigs, providing a time‐sensitive measure. A number of methods are available to detect porcine cysticercosis directly or indirectly; however, they vary greatly in their sensitivity, specificity and the quality of the evidence available to support diagnostic performance characteristics that have been claimed.

### Direct detection of cysticerci

Cysticerci can be detected in pigs either by palpation of the tongue or inspection of carcase tissues. Relatively few studies have undertaken tongue palpation before carcase examination, such that scientific data on specificity of tongue palpation are scarce; however, it has been reported to be high 66, 67. Tongue palpation is the simplest method for the detection of porcine cysticercosis. Estimates of the sensitivity of tongue palpation vary widely, from as low as 16% in some instances 66 to 70% 67. Sciutto *et al*. 68 did not undertake tongue inspection *per se*, but found only one of 18 naturally infected pigs that were subjected to full carcase dissection had any cysts in the tongue. Dorny *et al*. 69 failed to identify infection by tongue palpation in any of 10 pigs lightly infected with *T. solium* (<100 cysticerci) and detected infection in only five of 14 more heavily infected animals.

In pigs, *T. solium* encysts almost exclusively in the striated muscles and neural tissue 66. The definitive method for assessing the presence of cysticerci in pigs is by examination of carcase meat. Routine meat inspection as conducted in slaughterhouses is an insensitive procedure; as few as 38% of pigs infected with cysticerci were identified in Zambia 66 by meat inspection, even though the inspection procedure that was employed included both a rigorous examination of the carcase as well as incisions in the external and internal masseters, heart, triceps and shoulder. Boa *et al*. 70 determined that only 10·6% of *T. solium* cysts that were present in pigs could be identified in the organs that were examined at meat inspection.

Manual slicing of all carcase meat, and the brain, at approximately 3‐ to 5‐mm intervals will identify pigs with even very light *T. solium* infections. It is impossible to know the actual sensitivity of full carcase dissection and meat slicing, but it is likely to be close to 100%. The major disadvantage of carcase dissection is the cost, both for purchase of the pigs themselves as well as the skilled staff time required to carry out the examinations. An alternative to full carcase dissection can be dissection of some proportion of the meat. Simple probability would dictate that some lightly infected carcases would not be identified. Vargas Mendez *et al*. 71 found no difference in *T. solium* encystation rates between the left and right side of pig carcases. Examination of half, or even a lesser proportion of the carcase, would enhance the feasibility of carcase examination for determining the prevalence of porcine cysticercosis in comparison to full carcase dissection.

A number of studies have investigated the distribution of *T. solium* cysticerci in naturally or experimentally infected pigs 68, 70, 71, 72, 73. Sites at which cysts are more commonly located, or predilection sites, include the masseter muscles, pterygoid, tongue, heart and triceps. Sciutto *et al*. 68 found that 29 of 41 (71%) pigs experimentally infected with *T. solium* had cysts in the tongue (detected by slicing). However until recently, all publications describing the distribution of cysts in naturally infected animals have utilized pigs that were preselected as being infected with *T. solium* by tongue inspection. This is likely to have biased the sampling in favour of more heavily infected animals. Lightowlers *et al*. 74 analysed the *T. solium* cyst distribution data from extensive necropsy examinations undertaken on 209 unselected pigs obtained from rural regions in which *T. solium* was endemic. These pigs were control animals involved in TSOL18 vaccine field trials undertaken in Cameroon (102 pigs) 14 and Peru (107 pigs) 75. Of the animals that were detected as having *T. solium* infection (38 were infected), 81% were found to have cysts when only the heart, tongue and masticatory muscles were dissected. These data indicate that porcine cysticercosis can be diagnosed specifically and with a relatively high sensitivity by dissection of a limited but specific proportion of the muscle tissue. The proportion of infected pigs that were found to have cysts detected in the selected muscle sites was higher in animals having a heavier overall burden of infection. All the infected animals not found to have any cysts in the heart, tongue or masticatory muscles had relatively light infections (<50 cysts, mean = 15, median = 7). Whether the value of partial carcase dissection for diagnosis of porcine cysticercosis would be affected following implementation of control measures for *T. solium*, would depend on the effect that the intervention had on the average burden of infection in infected pigs. Considerable evidence indicates that much of the transmission of *T. solium* to pigs occurs focally near the residence of *T. solium* patients with taeniasis 76, 77, although not all studies have supported this conclusion 78. Should this be the case, changes in the prevalence of taeniasis might be expected to affect the incidence of porcine cysticercosis, but not necessarily the burden of parasites in infected animals.

### Serological tests

By far the simplest and least expensive method to diagnose porcine cysticercosis would be serology. Several tests have been developed and applied in many published studies on *T. solium* epidemiology or for assessment of *T. solium* control activities. Commercial kits are available for the detection of *T. solium* circulating antigens or anti‐*T. solium* antibodies in pigs. The available circulating antigen detection test is the same as that used for cysticercosis in humans. Purchase costs for the kit would entail a cost of €3·15 or USD 3·5/duplicate test. Quoted purchase costs for commercial antibody detection tests vary from USD 5–21 per test. While there is good evidence available to support the sensitivity of some tests, none have reported satisfactory evidence to substantiate claims concerning specificity.

Most of the serological tests that have been applied to porcine cysticercosis were developed initially for diagnosis of human cysticercosis. Some tests have been considered to be highly specific for *T. solium* cysticercosis, and the evidence to support the tests’ specificity in humans appears to be solid. However, pigs are known to be infected with, and are likely to be exposed to many more species of taeniid cestode parasite than are humans, due to their foraging habit. For this reason, the potential for nonspecific positive serological reactions in porcine *T. solium* serology is greater than in human cysticercosis serology. Coprophagia by pigs is well recognized, both in relation to human faeces and the faeces of other animals 79, 80, 81. In rural areas, dogs and other carnivores are frequently infected with *Taenia* species tapeworms, including in regions where *T. solium* is prevalent 82, 83, 84, 85. One of these species is known to infect pigs with viable cysticerci (*T. hydatigena*); however, there is potential for eggs of the other *Taenia* species to hatch if ingested by pigs, for the oncospheres to penetrate and transiently invade the tissues, possibly remaining for long enough to cause a transient antibody response or the presence of circulating antigen that could be detected by serological tests used for porcine cysticercosis.

It has been known for some time that many serologically positive pigs are found to have no cysts when necropsied 68, 86, 87, 88. More recent and comprehensive data have confirmed that the great majority of rural pigs that are serologically positive for porcine cysticercosis are found to have no cysticerci at necropsy 36, 89, 90. In studies where sequential serum samples have been obtained from pigs in *T. solium* endemic rural areas prior to them being necropsied and found to have no cysts, the animals were commonly shown to seroconvert from negative to positive and back to negative 36, 90.

It is possible that false‐positive/transient positive reactions in serological tests for porcine cysticercosis could be due to exposure of the animals to *T. solium* eggs which did not lead to the establishment of cysticerci that could be found at necropsy. In the case of tests known to cross‐react with *T. hydatigena*, an equally valid argument could be made to suggest that the transient positives were due to exposure to that parasite. *T. hydatigena* is known to be highly prevalent in some areas of Africa where *T. solium* is also endemic 91. In addition, no assessment has been made of the potential for cross‐reactivity in tests for porcine cysticercosis due to exposure to *T. saginata*. *T. saginata* is known to be capable of establishing viable cysticerci in the liver of pigs following experimental infection 92. *T. solium* and *T. saginata* are commonly co‐endemic. In Africa, even in areas where *T. solium* is highly prevalent and beef is rarely eaten such as the eastern Province of Zambia 60, *T. saginata* prevalence may contribute as much as 14% of patients with taeniasis. In other regions of Africa, such as in the Dodoma region of Tanzania, the prevalence of *T. saginata* taeniasis is substantially higher than *T. solium* 93. It seems unlikely that infections with *T. saginata* occur commonly in pig livers. Few publications have described examination of the livers of pigs from *T. solium* endemic areas, but those that have, did not identify cysticerci in the liver 70. Nevertheless, the common occurrence of human taeniasis due to *T. saginata* in many areas where *T. solium* also occurs suggests that pigs have ample opportunity to be exposed to *T. saginata* eggs, in some situations more frequently than they are exposed to *T. solium*. Transient positive reactivity in serological tests for porcine cysticercosis could be partially or entirely due to exposure to *T. saginata*.

Pigs experimentally infected with *T. asiatica* show seroconversion within one week of infection in one antigen ELISA test commonly used for *T. solium* diagnosis 37. Seroconversion occurs long before the development of mature cysticerci, emphasizing the potential for exposure to *T. saginata* to lead to false‐positive reactions in serological tests for porcine cysticercosis. Pigs exposed to *T. asiatica* are also positive in the EITB assay 26 that has been used frequently for diagnosis of both porcine and human cysticercosis.

Until data are available about serological responses in pigs following exposure to a variety of *Taenia* species, particularly *T. hydatigena* and *T. saginata*, none of the tests currently being employed can be considered to have been adequately assessed for specificity. Data published to date using porcine cysticercosis serology need to be interpreted with caution. Nevertheless, while the specificity of both EITB and antigen ELISA tests for porcine cysticercosis require further evaluation or improvement, both tests have a relatively high sensitivity for the detection of infection in most infected pigs. Hence, the tests have potential value in screening and selecting animals for necropsy. In this way, many uninfected animals could reasonably be excluded from requiring necropsy.

### Research/development needs

- Further evidence is required concerning the specificity of all serological methods that are currently used for porcine cysticercosis.
- Improvement in the specificity of the antigen ELISA, certainly in relation to cross‐reactivity with *T. hydatigena*, but possibly also in relation to other possible causes of nonspecific reactions.
- Further validation of the sensitivity of examination of only a small part of pig carcases to achieve a relatively high sensitivity for diagnosis of infection in unselected, naturally infected pigs.

## Influence of Sample Size on Monitoring *Taenia solium* Interventions

**Figure 1:**
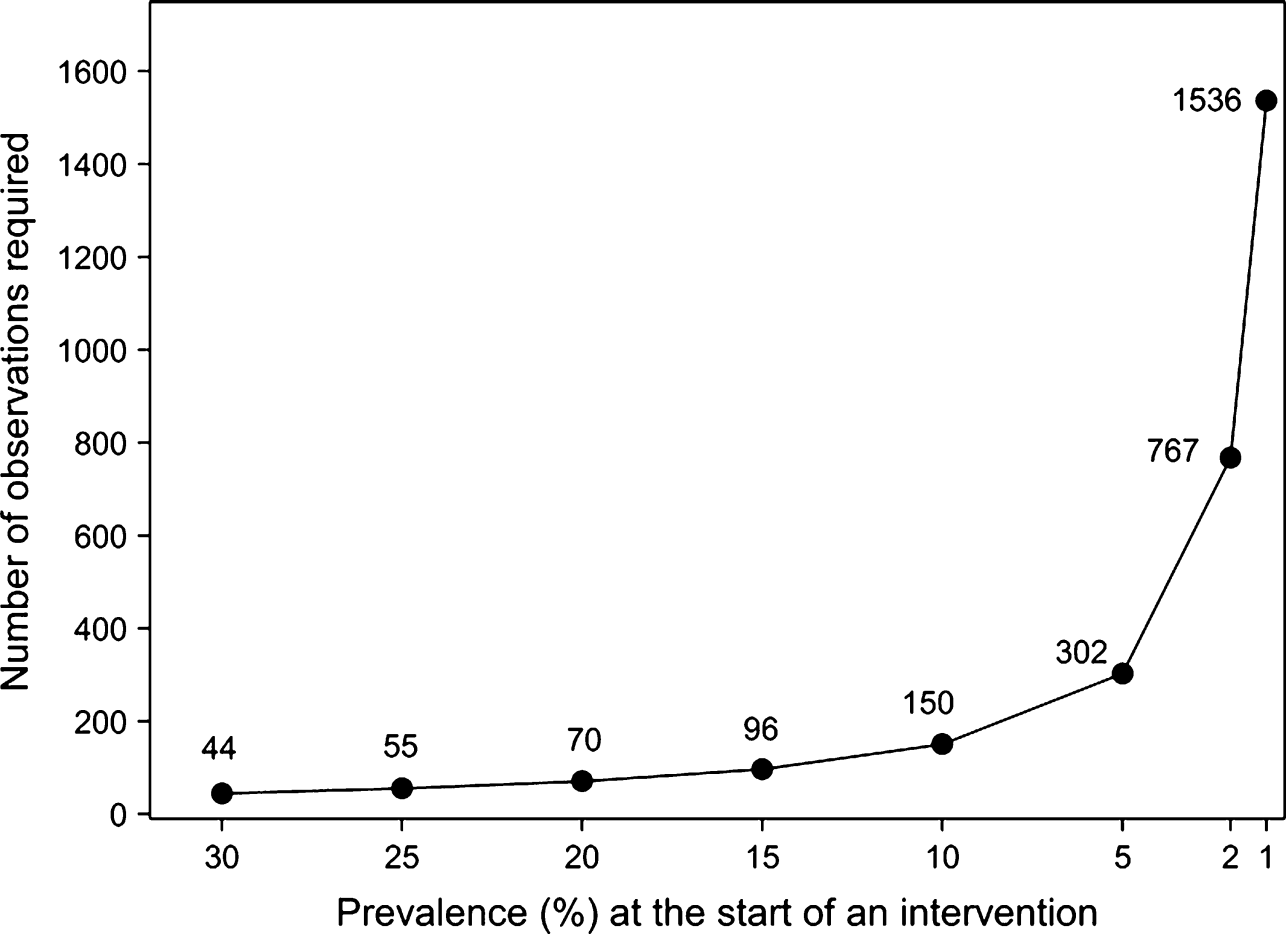
Number of observations required at the start and at the end of an intervention period in order to detect an 80% decrease in prevalence with a type I error of 5% and a power of 80% (Fisher's exact test, two sided). The numbers of observations are shown for each prevalence value plotted.

Comments have been made above about the impact that a low overall prevalence of human taeniasis or cysticercosis would have on sample size requirements if these measures were used to evaluate the outcomes of *T. solium* control initiatives. While it would be important, and difficult, to minimize sample biasing while attempting to quantify the prevalence of taeniasis or cysticercosis, it is possible to determine the sample sizes that would be required to be evaluated assuming that sample bias could be avoided. Figure 1 illustrates the number of observations that would be required to be made both at the start and at the end of an intervention period in order to detect an 80% decrease in infection prevalence with a type I error of 5% and a power of 80%. Where the prevalence of a particular assessment measure was relatively high at the beginning of an intervention, a relatively small number of samples would be required to be taken to detect a significant change due to intervention activities. While the precise numbers illustrated in Figure 1 may differ slightly according to the particular assumptions used in the analyses, an example obtained using Fisher's exact test indicates that where there was a 25% prevalence of pig cysticercosis, the number of samples required to be able to detect at least an 80% decrease in porcine cysticercosis would be 55, taken at both the start and the end of the intervention. However, where the prevalence of the measure being evaluated was rare, for example a 2% prevalence of taeniasis, samples of 767 at both the start and end of an intervention would be required to detect an 80% decline over the period of the intervention.

## Suggestions for Monitoring Future *Taenia Solium* Control Initiatives

Based on the currently available data, and for the reasons elaborated above, the following suggestions are made about the most effective and appropriate methods that can be used for monitoring future *T. solium* control initiatives.

### Human cysticercosis

- Assessment of human cysticercosis levels as part of a *T. solium* control initiative would only be recommended where the sample size that could be evaluated was commensurate with obtaining statistically significant results.
- The use of serological tests which simply determine seroconversion cannot be recommended to evaluate control interventions until further data are available to confirm that transient serologically positive responses are uniquely due to exposure to *T. solium* and not some other cause. Measurements of prevalence using serological methods cannot be recommended because of the persistence of positive reactions in cysticercotic patients over the period of the intervention. Measures of incidence could be used, as determined by either EITB or antigen ELISA, provided the new cases were shown to remain persistently positive (i.e. are likely cases of actual infection).

### Taeniasis

- Assessment of taeniasis levels as part of a *T. solium* control initiative would only be recommended where the sample size that could be evaluated was commensurate with obtaining statistically significant results.
- The most effective and suggested method for species‐specific detection of human *T. solium* taeniasis is the nested PCR method developed by Mayta *et al*. 57.

### Porcine cysticercosis

- Serological methods cannot be recommended for porcine cysticercosis because none of the methods available has been adequately assessed for specificity. Due to their high sensitivity for detecting porcine cysticercosis, serological methods could be used as a pre‐necropsy screen so as to eliminate the need to undertake necropsy on uninfected animals.
- Porcine cysticercosis can only be adequately assessed by slicing carcase meat at necropsy. Thorough slicing of the heart, tongue and masticatory muscles is sufficient to detect 80% of naturally infected pigs and, where resources would not allow examination of more or all carcase meat and brain, assessment of the heart, tongue and masticatory muscles alone is suggested for diagnosis of porcine cysticercosis.

## References

[1] Garcia HH & Del Brutto OH . Neurocysticercosis: updated concepts about an old disease. Lancet Neurol 2005; 4: 653–661. 16168934 10.1016/S1474-4422(05)70194-0

[2] Mahanty S , Garcia HH & Cysticercosis Working Group in Peru . Cysticercosis and neurocysticercosis as pathogens affecting the nervous system. Prog Neurobiol 2010; 91: 172–184. 20035822 10.1016/j.pneurobio.2009.12.008

[3] Del Brutto OH & Garcia HH . Neurocysticercosis in nonendemic countries: time for a reappraisal. Neuroepidemiology 2012; 39: 145–146. 22922488 10.1159/000341693

[4] Garcia HH & Cysticercosis Working Group in Peru . Neurocysticercosis in immigrant populations. J Travel Med 2012; 19: 73–75. 22414030 10.1111/j.1708-8305.2011.00583.x PMC3349430

[5] Ndimubanzi PC , Carabin H , Budke CM , *et al* A systematic review of the frequency of neurocyticercosis with a focus on people with epilepsy. PLoS Negl Trop Dis 2010; 4: e870. 21072231 10.1371/journal.pntd.0000870 PMC2970544

[6] Robertson LJ , van der Giessen JW , Batz MB , Kojima M & Cahill S . Have foodborne parasites finally become a global concern? Trends Parasitol 2013; 29: 101–103. 23375923 10.1016/j.pt.2012.12.004

[7] World Health Organization . Investing to overcome the global impact of Neglected Tropical Diseases. Third WHO report on neglected tropical diseases. WHO/HTM/NTD/2015.1Geneva; 2015.

[8] Pawlowski Z , Allan J & Sarti E . Control of *Taenia solium* taeniasis/cysticercosis: from research towards implementation. Int J Parasitol 2005; 35: 1221–1232. 16143335 10.1016/j.ijpara.2005.07.015

[9] Lightowlers MW . Control of *Taenia solium* taeniasis/cysticercosis: past practices and new possibilities. Parasitology 2013; 140: 1566–1577. 23947762 10.1017/S0031182013001005

[10] Allan JC & Avila G , Garcia Noval J , Flisser A & Craig PS . Immunodiagnosis of taeniasis by coproantigen detection. Parasitology 1990; 101: 473–477. 2092303 10.1017/s0031182000060686

[11] Gonzales AE , Garcia HH , Gilman RH , *et al* Effective, single‐dose treatment of porcine cysticercosis with oxfendazole. Am J Trop Med Hyg 1996; 54: 391–394. 8615453 10.4269/ajtmh.1996.54.391

[12] Flisser A , Gauci CG , Zoli A , *et al* Induction of protection against porcine cysticercosis by vaccination with recombinant oncosphere antigens. Infect Immun 2004; 72: 5292–5297. 15322025 10.1128/IAI.72.9.5292-5297.2004 PMC517464

[13] Gonzalez AE , Gauci CG , Barber D , *et al* Vaccination of pigs to control human neurocysticercosis. Am J Trop Med Hyg 2005; 72: 837–839. 15964973

[14] Assana E , Kyngdon CT , Gauci CG , *et al* Elimination of *Taenia solium* transmission to pigs in a field trial of the TSOL18 vaccine in Cameroon. Int J Parasitol 2010; 40: 515–519. 20138046 10.1016/j.ijpara.2010.01.006 PMC2856920

[15] Garcia HH , Rodriguez S , Gilman RH , Gonzalez AE , Tsang VC & Cysticercosis Working Group in Peru . Neurocysticercosis: is serology useful in the absence of brain imaging? Trop Med Int Health 2012; 17: 1014–1018. 22809375 10.1111/j.1365-3156.2012.03037.x

[16] Rodriguez S , Wilkins P & Dorny P . Immunological and molecular diagnosis of cysticercosis. Pathog Glob Health 2012; 106: 286–298. 23265553 10.1179/2047773212Y.0000000048 PMC4005112

[17] Parkhouse RM & Harrison LJ . Cyst fluid and surface associated glycoprotein antigens of *Taenia* sp. metacestodes. Parasite Immunol 1987; 9: 263–268. 3574977 10.1111/j.1365-3024.1987.tb00505.x

[18] Tsang VC , Brand JA & Boyer AE . An enzyme‐linked immunoelectrotransfer blot assay and glycoprotein antigens for diagnosing human cysticercosis (*Taenia solium*). J Infect Dis 1989; 159: 50–59. 2909643 10.1093/infdis/159.1.50

[19] Noh J , Rodriguez S , Lee YM , *et al* Recombinant protein‐ and synthetic peptide‐based immunoblot test for diagnosis of neurocysticercosis. J Clin Microbiol 2014; 52: 1429–1434. 24554747 10.1128/JCM.03260-13 PMC3993633

[20] Scheel CM , Khan A , Hancock K , *et al* Serodiagnosis of neurocysticercosis using synthetic 8‐kD proteins: comparison of assay formats. Am J Trop Med Hyg 2005; 73: 771–776. 16222024

[21] Bueno EC , Scheel CM , Vaz AJ , *et al* Application of synthetic 8‐kD and recombinant GP50 antigens in the diagnosis of neurocysticercosis by enzyme‐linked immunosorbent assay. Am J Trop Med Hyg 2005; 72: 278–283. 15772321

[22] Ito A , Plancarte A , Ma L , *et al* Novel antigens for neurocysticercosis: simple method for preparation and evaluation for serodiagnosis. Am J Trop Med Hyg 1998; 59: 291–294. 9715949 10.4269/ajtmh.1998.59.291

[23] Garcia HH , Gonzalez AE , Gilman RH , *et al* Short report: transient antibody response in *Taenia solium* infection in field conditions‐a major contributor to high seroprevalence. Am J Trop Med Hyg 2001; 65: 31–32. 11504404 10.4269/ajtmh.2001.65.31

[24] Meza‐Lucas A , Carmona‐Miranda L , Garcia‐Jeronimo RC , *et al* Short report: limited and short‐lasting humoral response in *Taenia solium*: seropositive households compared with patients with neurocysticercosis. Am J Trop Med Hyg 2003; 69: 223–227. 13677380

[25] Mwape KE , Phiri IK , Praet N , *et al* The incidence of human cysticercosis in a rural community of Eastern Zambia. PLoS Negl Trop Dis 2013; 7: e2142. 23556026 10.1371/journal.pntd.0002142 PMC3605208

[26] Pilcher JB , Tsang VC , Gilman RH , Rhodes ML & Pawlowski ZS . Further evidence of 100% specificity in a recently developed *Taenia solium* (cysticercosis) immunoblot assay. Am J Trop Med Hyg 1991; 45(suppl 3): 131.

[27] Harrison LJ , Joshua GW , Wright SH & Parkhouse RM . Specific detection of circulating surface/secreted glycoproteins of viable cysticerci in *Taenia saginata* cysticercosis. Parasite Immunol 1989; 11: 351–370. 2674862 10.1111/j.1365-3024.1989.tb00673.x

[28] Brandt JR , Geerts S , De Deken R , *et al* A monoclonal antibody‐based ELISA for the detection of circulating excretory‐secretory antigens in *Taenia saginata* cysticercosis. Int J Parasitol 1992; 22: 471–477. 1644522 10.1016/0020-7519(92)90148-e

[29] Van Kerckhoven I , Vansteenkiste W , Claes M , Geerts S & Brandt J . Improved detection of circulating antigen in cattle infected with *Taenia saginata* metacestodes. Vet Parasitol 1998; 76: 269–274. 9650864 10.1016/s0304-4017(97)00226-4

[30] Rodriguez‐del‐Rosal E , Correa D & Flisser A . Swine cysticercosis: detection of parasite products in serum. Vet Rec 1989; 124: 488. 2665310 10.1136/vr.124.18.488

[31] Nguekam A , Zoli AP , Vondou L , *et al* Kinetics of circulating antigens in pigs experimentally infected with *Taenia solium* eggs. Vet Parasitol 2003; 111: 323–332. 12559711 10.1016/s0304-4017(02)00391-6

[32] Correa D , Sandoval MA , Harrison LJ , *et al* Human neurocysticercosis: comparison of enzyme immunoassay capture techniques based on monoclonal and polyclonal antibodies for the detection of parasite products in cerebrospinal fluid. Trans R Soc Trop Med Hyg 1989; 83: 814–816. 2694513 10.1016/0035-9203(89)90340-4

[33] Erhart A , Dorny P , Van De N , *et al* *Taenia solium* cysticercosis in a village in northern Viet Nam: seroprevalence study using an ELISA for detecting circulating antigen. Trans R Soc Trop Med Hyg 2002; 96: 270–272. 12174775 10.1016/s0035-9203(02)90095-7

[34] Garcia HH , Parkhouse RM , Gilman RH , *et al* Serum antigen detection in the diagnosis, treatment, and follow‐up of neurocysticercosis patients. Trans R Soc Trop Med Hyg 2000; 94: 673–676. 11198654 10.1016/s0035-9203(00)90228-1

[35] Coral‐Almeida M , Rodriguez‐Hidalgo R , Celi‐Erazo M , *et al* Incidence of human *Taenia solium* larval Infections in an Ecuadorian endemic area: implications for disease burden assessment and control. PLoS Negl Trop Dis 2014; 8: e2887. 24852050 10.1371/journal.pntd.0002887 PMC4031064

[36] Devleesschauwer B , Aryal A , Tharmalingam J , *et al* Complexities in using sentinel pigs to study *Taenia solium* transmission dynamics under field conditions. Vet Parasitol 2013; 193: 172–178. 23298565 10.1016/j.vetpar.2012.12.010

[37] Geerts S , Zorloni A , Kumar V , Brandt JR , De Deken R & Eom KS . Experimental infection of pigs with a *Taenia* species from Korea: parasitological and serological aspects. Parasitol Res 1992; 78: 513–515. 1438138 10.1007/BF00931573

[38] Okello A , Ash A , Keokhamphet C , *et al* Investigating a hyper‐endemic focus of *Taenia solium* in northern Lao PDR. Parasit Vectors 2014; 7: 134. 24678662 10.1186/1756-3305-7-134 PMC3986602

[39] Mwanjali G , Kihamia C , Kakoko DV , *et al* Prevalence and risk factors associated with human *Taenia solium* infections in Mbozi District, Mbeya Region, Tanzania. PLoS Negl Trop Dis 2013; 7: e2102. 23516650 10.1371/journal.pntd.0002102 PMC3597471

[40] Li T , Chen X , Yanagida T , *et al* Detection of human taeniases in Tibetan endemic areas, China. Parasitology 2013; 140: 1602–1607. 23866973 10.1017/S003118201300111X

[41] Jeon HK , Yong TS , Sohn WM , *et al* Current status of human taeniasis in Lao People's Democratic Republic. Korean J Parasitol 2013; 51: 259–263. 23710098 10.3347/kjp.2013.51.2.259 PMC3662074

[42] Watts NS , Pajuelo M , Clark T , *et al* *Taenia solium* infection in Peru: a collaboration between Peace Corps Volunteers and researchers in a community based study. PLoS ONE 2014; 9: e113239. 25469506 10.1371/journal.pone.0113239 PMC4254459

[43] Pawlowski Z & Schultz MG . Taeniasis and cysticercosis (*Taenia saginata*). Adv Parasitol 1972; 10: 269–343. 4559145 10.1016/s0065-308x(08)60176-1

[44] Hall A , Latham MC , Crompton DW & Stephenson LS . *Taenia saginata* (Cestoda) in western Kenya: the reliability of faecal examinations in diagnosis. Parasitology 1981; 83: 91–101. 7196568 10.1017/s003118200005006x

[45] Allan JC , Velasquez‐Tohom M , Torres‐Alvarez R , Yurrita P & Garcia‐Noval J . Field trial of the coproantigen‐based diagnosis of *Taenia solium* taeniasis by enzyme‐linked immunosorbent assay. Am J Trop Med Hyg 1996; 54: 352–356. 8615446 10.4269/ajtmh.1996.54.352

[46] Guezala MC , Rodriguez S , Zamora H , *et al* Development of a species‐specific coproantigen ELISA for human *Taenia solium* taeniasis. Am J Trop Med Hyg 2009; 81: 433–437. 19706909

[47] Bustos JA , Rodriguez S , Jimenez JA , *et al* Detection of *Taenia solium* taeniasis coproantigen is an early indicator of treatment failure for taeniasis. Clin Vaccine Immunol 2012; 19: 570–573. 22336287 10.1128/CVI.05428-11 PMC3318286

[48] Tembo A & Craig PS . *Taenia saginata* taeniosis: copro‐antigen time‐course in a voluntary self‐infection. J Helminthol 2015; 89: 612–619. 24945107 10.1017/S0022149X14000455

[49] Jenkins DJ & Rickard MD . Specific antibody responses to *Taenia hydatigena*,* Taenia pisiformis* and *Echinococcus granulosus* infection in dogs. Aust Vet J 1985; 62: 72–78. 4015557 10.1111/j.1751-0813.1985.tb14142.x

[50] Heath DD , Lawrence SB , Glennie A & Twaalfhoven H . The use of excretory and secretory antigens of the scolex of *Taenia ovis* for the serodiagnosis of infection in dogs. J Parasitol 1985; 71: 192–199. 3998957

[51] Wilkins PP , Allan JC , Verastegui M , *et al* Development of a serologic assay to detect *Taenia solium* taeniasis. Am J Trop Med Hyg 1999; 60: 199–204. 10072136 10.4269/ajtmh.1999.60.199

[52] Levine MZ , Lewis MM , Rodriquez S , *et al* Development of an enzyme‐linked immunoelectrotransfer blot (EITB) assay using two baculovirus expressed recombinant antigens for diagnosis of *Taenia solium* taeniasis. J Parasitol 2007; 93: 409–417. 17539427 10.1645/GE-938R.1

[53] Levine MZ , Calderon JC , Wilkins PP , *et al* Characterization, cloning, and expression of two diagnostic antigens for *Taenia solium* tapeworm infection. J Parasitol 2004; 90: 631–638. 15270112 10.1645/GE-189R

[54] Handali S , Klarman M , Gaspard AN , *et al* Multiantigen print immunoassay for comparison of diagnostic antigens for *Taenia solium* cysticercosis and taeniasis. Clin Vaccine Immunol 2010; 17: 68–72. 19906893 10.1128/CVI.00339-09 PMC2812080

[55] Handali S , Klarman M , Gaspard AN , *et al* Development and evaluation of a magnetic immunochromatographic test to detect *Taenia solium*, which causes taeniasis and neurocysticercosis in humans. Clin Vaccine Immunol 2010; 17: 631–637. 20181766 10.1128/CVI.00511-09 PMC2849338

[56] Verweij JJ & Stensvold CR . Molecular testing for clinical diagnosis and epidemiological investigations of intestinal parasitic infections. Clin Microbiol Rev 2014; 27: 371–418. 24696439 10.1128/CMR.00122-13 PMC3993103

[57] Mayta H , Gilman RH , Prendergast E , *et al* Nested PCR for specific diagnosis of *Taenia solium* taeniasis. J Clin Microbiol 2008; 46: 286–289. 17989190 10.1128/JCM.01172-07 PMC2224258

[58] Nkouawa A , Sako Y , Li T , *et al* Evaluation of a loop‐mediated isothermal amplification method using fecal specimens for differential detection of *Taenia* species from humans. J Clin Microbiol 2010; 48: 3350–3352. 20631114 10.1128/JCM.00697-10 PMC2937673

[59] Yamasaki H , Allan JC , Sato MO , *et al* DNA differential diagnosis of taeniasis and cysticercosis by multiplex PCR. J Clin Microbiol 2004; 42: 548–553. 14766815 10.1128/JCM.42.2.548-553.2004 PMC344500

[60] Praet N , Verweij JJ , Mwape KE , *et al* Bayesian modelling to estimate the test characteristics of coprology, coproantigen ELISA and a novel real‐time PCR for the diagnosis of taeniasis. Trop Med Int Health 2013; 18: 608–614. 23464616 10.1111/tmi.12089

[61] Nunes CM , Lima LG , Manoel CS , Pereira RN , Nakano MM & Garcia JF . *Taenia saginata*: polymerase chain reaction for taeniasis diagnosis in human fecal samples. Exp Parasitol 2003; 104: 67–69. 12932762 10.1016/s0014-4894(03)00111-5

[62] Nunes CM , Lima LG , Manoel CS , Pereira RN , Nakano MM & Garcia JF . Fecal specimens preparation methods for PCR diagnosis of human taeniosis. Rev Inst Med Trop Sao Paulo 2006; 48: 45–47. 16547580 10.1590/s0036-46652006000100010

[63] Nkouawa A , Sako Y , Li T , *et al* A loop‐mediated isothermal amplification method for a differential identification of *Taenia* tapeworms from human: application to a field survey. Parasitol Int 2012; 61: 723–725. 22698671 10.1016/j.parint.2012.06.001

[64] Ito A , Putra MI , Subahar R , *et al* Dogs as alternative intermediate hosts of *Taenia solium* in Papua (Irian Jaya), Indonesia confirmed by highly specific ELISA and immunoblot using native and recombinant antigens and mitochondrial DNA analysis. J Helminthol 2002; 76: 311–314. 12498635 10.1079/JOH2002128

[65] Fan PC , Chung WC & Wu JC . Experimental infection of an isolate of *Taenia solium* from Hainan in domestic animals. J Helminthol 1994; 68: 265–266. 7829849 10.1017/s0022149x00014450

[66] Phiri IK , Dorny P , Gabriel S , *et al* Assessment of routine inspection methods for porcine cysticercosis in Zambian village pigs. J Helminthol 2006; 80: 69–72. 16469176 10.1079/joh2005314

[67] Gonzalez AE , Cama V , Gilman RH , *et al* Prevalence and comparison of serologic assays, necropsy, and tongue examination for the diagnosis of porcine cysticercosis in Peru. Am J Trop Med Hyg 1990; 43: 194–199. 2389823 10.4269/ajtmh.1990.43.194

[68] Sciutto E , Martinez JJ , Villalobos NM , *et al* Limitations of current diagnostic procedures for the diagnosis of *Taenia solium* cysticercosis in rural pigs. Vet Parasitol 1998; 79: 299–313. 9831953 10.1016/s0304-4017(98)00180-0

[69] Dorny P , Phiri IK , Vercruysse J , *et al* A Bayesian approach for estimating values for prevalence and diagnostic test characteristics of porcine cysticercosis. Int J Parasitol 2004; 34: 569–576. 15064121 10.1016/j.ijpara.2003.11.014

[70] Boa ME , Kassuku AA , Willingham AL III , Keyyu JD , Phiri IK & Nansen P . Distribution and density of cysticerci of *Taenia solium* by muscle groups and organs in naturally infected local finished pigs in Tanzania. Vet Parasitol 2002; 106: 155–164. 12031817 10.1016/s0304-4017(02)00037-7

[71] Vargas Mendez GD , Saldierna U , Navarro Fierro R , Acevedo Hernandez A , de Flisser MA & Aluja ASd . Localizacion del cisticerco de la *Taenia solium* en diferentes regiones musculares del cerdo y su importancia para la inspeccion sanitaria [Localization of *Taenia solium* cysticerci in different muscular regions of swine and its significance in meat inspection]. Vet Mexico 1986; 17: 275–279.

[72] Sikasunge CS , Johansen MV , Willingham AL III , Leifsson PS & Phiri IK . *Taenia solium* porcine cysticercosis: viability of cysticerci and persistency of antibodies and cysticercal antigens after treatment with oxfendazole. Vet Parasitol 2008; 158: 57–66. 18834668 10.1016/j.vetpar.2008.08.014

[73] Gonzalez AE , Falcon N , Gavidia C , *et al* Time‐response curve of oxfendazole in the treatment of swine cysticercosis. Am J Trop Med Hyg 1998; 59: 832–836. 9840607 10.4269/ajtmh.1998.59.832

[74] Lightowlers MW , Assana E , Jayashi CM , Gauci CG & Donadeu M . Sensitivity of partial carcass dissection for assessment of porcine cysticercosis at necropsy. Int J Parasitol 2015; 45: 815–818. 26385439 10.1016/j.ijpara.2015.08.004 PMC4655835

[75] Jayashi CM , Kyngdon CT , Gauci CG , Gonzalez AE & Lightowlers MW . Successful immunization of naturally reared pigs against porcine cysticercosis with a recombinant oncosphere antigen vaccine. Vet Parasitol 2012; 188: 261–267. 22541797 10.1016/j.vetpar.2012.03.055 PMC3420019

[76] Lescano AG , Garcia HH , Gilman RH , *et al* Swine cysticercosis hotspots surrounding *Taenia solium* tapeworm carriers. Am J Trop Med Hyg 2007; 76: 376–383. 17297051

[77] O'Neal SE , Moyano LM , Ayvar V , *et al* Geographic correlation between tapeworm carriers and heavily infected cysticercotic pigs. PLoS Negl Trop Dis 2012; 6: e1953. 23285305 10.1371/journal.pntd.0001953 PMC3527375

[78] Morales J , Martinez JJ , Rosetti M , *et al* Spatial distribution of *Taenia solium* porcine cysticercosis within a rural area of Mexico. PLoS Negl Trop Dis 2008; 2: e284. 18846230 10.1371/journal.pntd.0000284 PMC2565694

[79] Copado F , de Aluja AS , Mayagoitia L & Galindo F . The behaviour of free ranging pigs in the Mexican tropics and its relationships with human faeces consumption. Appl Anim Behav Sci 2004; 88: 243–252.

[80] Soave O & Brand CD . Coprophagy in animals: a review. Cornell Vet 1991; 81: 357–364. 1954740

[81] de Aluja A Frequency of porcine cysticercosis in Mexico In Flisser A, Willms K, Laclette JP, Larralde C, Ridaura C, Beltran F (eds): Cysticercosis: Present State of Knowledge and Perspectives. New York, Academic Press, 1982: 53–62.

[82] Berentsen AR , Becker MS , Stockdale‐Walden H , Matandiko W , McRobb R & Dunbar MR . Survey of gastrointestinal parasite infection in African lion (*Panthera leo*), African wild dog (*Lycaon pictus*) and spotted hyaena (*Crocuta crocuta*) in the Luangwa Valley, Zambia. Afr Zool 2012; 47: 363–368.

[83] Nonaka N , Nakamura S , Inoue T , *et al* Coprological survey of alimentary tract parasites in dogs from Zambia and evaluation of a coproantigen assay for canine echinococcosis. Ann Trop Med Parasitol 2011; 105: 521–530. 22185947 10.1179/2047773211Y.0000000001 PMC4100310

[84] Conlan JV , Vongxay K , Khamlome B , *et al* A cross‐sectional study of *Taenia solium* in a multiple taeniid‐endemic region reveals competition may be protective. Am J Trop Med Hyg 2012; 87: 281–291. 22855759 10.4269/ajtmh.2012.11-0106 PMC3414565

[85] Verster A . Gastrointestinal helminths of domestic dogs in the Republic of South Africa. Onderstepoort J Vet Res 1979; 46: 79–82. 575920

[86] D'Souza PE & Hafeez M . Detection of *Taenia solium* cysticercosis in pigs by ELISA with an excretory‐secretory antigen. Vet Res Commun 1999; 23: 293–298. 10493116 10.1023/a:1006366920111

[87] Sciutto E , Hernandez M , Garcia G , *et al* Diagnosis of porcine cysticercosis: a comparative study of serological tests for detection of circulating antibody and viable parasites. Vet Parasitol 1998; 78: 185–194. 9760060 10.1016/s0304-4017(98)00129-0

[88] Gonzalez AE , Gilman R , Garcia HH , *et al* Use of sentinel pigs to monitor environmental *Taenia solium* contamination. The Cysticercosis Working Group in Peru (CWG). Am J Trop Med Hyg 1994; 51: 847–850. 7810821 10.4269/ajtmh.1994.51.847

[89] Gavidia CM , Verastegui MR , Garcia HH , *et al* Relationship between serum antibodies and *Taenia solium* larvae burden in pigs raised in field conditions. PLoS Negl Trop Dis 2013; 7: e2192. 23658848 10.1371/journal.pntd.0002192 PMC3642188

[90] Jayashi CM , Gonzalez AE , Neyra RC , Rodriguez S , Garcia HH & Lightowlers MW . Validity of the enzyme‐linked immunoelectrotransfer blot (EITB) for naturally acquired porcine cysticercosis. Vet Parasitol 2014; 199: 42–49. 24183647 10.1016/j.vetpar.2013.10.004 PMC5448663

[91] Braae UC , Kabululu M , Normark ME , Nejsum P , Ngowi HA & Johansen MV . *Taenia hydatigena* cysticercosis in slaughtered pigs, goats, and sheep in Tanzania. Trop Anim Health Prod 2015; DOI: 10.1007/s11250‐015‐0892‐6. in press. 10.1007/s11250-015-0892-6 26210397

[92] Fan PC , Chung WC , Lin CY & Wu CC . The pig as an intermediate host for Taiwan *Taenia* infection. J Helminthol 1990; 64: 223–231. 2230032 10.1017/s0022149x00012207

[93] Eom KS , Chai JY , Yong TS , *et al* Morphologic and genetic identification of *Taenia* tapeworms in Tanzania and DNA genotyping of *Taenia solium* . Korean J Parasitol 2011; 49: 399–403. 22355207 10.3347/kjp.2011.49.4.399 PMC3279678

